# Notes and News

**Published:** 1890-05-24

**Authors:** 


### Notes and News.

Collected and Communicated.

The annual report of the Mary Wardell Convalescent Home for scarlet fever says they had 211 inmates last year, and many male patients had to be refused for want of funds. There is a debt on the Home of £300, and £500 is badly needed for purchase of a steam disinfecting apparatus; so if anyone has too much money we recommend him to study this report.

Surgeon Parke is very much to the fore in society just now, and receives a hearty welcome and well earned applause wherever he goes. He was present at the Literary Fund dinner last week, and hurried away from there to attend the conversazione of the Royal Society. In the medical world there is more eagerness to see Surgeon Parke's book than Stanley's, but the explorer's will be produced first, and the doctor's some three weeks later.

It is our intention to issue, as usual, a special number for Hospital Sunday; this number will be published on May 31st, and will be distributed through the same channels as formerly. Some years ago when the *Lancet* stopped issuing a Hospital Sunday number we gladly threw ourselves into the breach, and having once undertaken this work we do not mean to drop it, though our contemporary has repented of the error of its ways, and once more announces a special number.

The last returns of the Metropolitan Asylums Board seem to indicate that fever is spreading in spite of the Notification of Diseases Act. The admissions of infectious cases into the managers' hospitals during the last three fortnights have been, on the one ending April 17th, 197; on that ending May 1st, 192; and on that ending the 15th of the present month, 278. In the report of the Eastern Hospital, of which Sir John Tilley is the chairman, a report was presented showing that there is a necessity for increasing the accommodation. It was shown that in 1872, 611 were all the cases admitted, while last year 2,091 were treated. The addition of diphtheric patients to the cases treated at the hospitals had added to the necessities for increased room.

The Lord Mayor on Tuesday carried out the promise he made at the Christmas gathering at the Evelina Hospital, and presided at a dinner at the Hotel Métropole in aid of that institution. The dinner proved a most enjoyable one, and a handsome subscription list was raised.

The Lord Mayor calls the attention of all ministers to the scheme by which two West-end churches raise such noble sums on Hospital Sunday, which is to be held on June 8th. The plan adopted by the ministers in both cases has been to sign circular letters to all the wealthier members of their congregation several days before Hospital Sunday, not only as a reminder of the day, but asking for liberal donations to help the sick poor, and also requesting all who cannot be present at Divine worship to send their contributions by post, so as to be added to the collections.

What a pity one of our illustrated papers does not do a round of hospital sketches. This is being done in Australia much to the edification of the public and the benefit of the institutions. In the *Illustrated Sydney News* for April 3, for instance, is a long account of the Coast Hospital, Little Bay. This is a hospital on the pavilion system chiefly for infectious cases, and lepers, lock cases and emergency cases are all admitted. The illustrations include a general view of the building, and portraits of the medical superintendent and the matron.

The Council of the Church of England Burial, Funeral, and Mourning Reform Association, at its late meeting, at the Church House, Westminster, resolved on petitioning the Home Secretary to issue an Order in Council prohibiting all burials in the catacombs at Brompton Cemetery. It was announced that the Duke of Westminster had contributed £100 to the funds of the society. A protest was uttered against excessive floral decorations at funerals. Mourners were recommended not to stand bareheaded for any length of time by the graveside, in the scorching sun or bitter cold.

The City of London Truss Society was instituted in the year 1807, and has already relieved nearly half a million of the suffering poor of both sexes and of all ages. The number for last year was 10,065. It is found that the proportion of aggravated cases is larger than it formerly was, and more expensive instruments are thus required, entailing a heavier expenditure on the society, and obliging it to seek for increased funds. The committee of the society earnestly appeal for further help for carrying on the work in which they are engaged, which is of vital importance to the labouring poor of the metropolis.

The opium trade in China and the liquor traffic among the natives of Natal are two subjects which should make the ears of Englishmen burn. Only a century and a quarter ago the consumption of opium in China was merely nominal, there being then little or no native cultivation, and the whole import of foreign opium being only about two hundred chests a year. In 1796 the Government, warned by the growing evil, forbade the trade. In 1839 Great Britain compelled China to permit the import in the interest of our Indian revenues, and now the annual importation from India is estimated at three hundred thousand chests. This is not the worst part of the business; for the removal of the interdict has led to a great extension of the native cultivation, and flourishing poppy-fields give mournful token of the progress of the opium-smoker's vice. The Governor of Natal says the nine thousand male Indians who have emigrated from Madras to Natal, consume eight gallons of rum for every man per annum. Evil accounts of the drink traffic reach us also from the Congo; the whole story is one of shame.

The following vacancies are announced this week, the amount of the salary in each case being given after the name of the institution:—

**Resident Medical Officers.**

Royal Westminster Ophthalmic Hospital, House Surgeon.

Central London Ophthalmic House Surgeon—Board, etc.

Shoreditch Infirmary, Medical Officer—£100, board, etc.

Wallasey Dispensary, House Surgeon—£80, rooms, etc.

Croydon General Hospital, House Surgeon—£100, board, etc.

Chichester Infirmary, House Surgeon—£80, board, etc.

Barnwood Hospital, Gloucester, Junior Assistant Medical Officer—£100, board, etc.

Brighton and Hove Dispensary, House Surgeon—£140, rooms, etc.

Mater Misericordiæ, Dublin, House Surgeon—£80, board, etc.

Norwich Friendly Societies, Resident Dispenser—£75.

**Non-Resident Medical Officers.**

Borough of Bolton, Medical Officer—£400.

Hartlepool Union, Medical Officer—£30.

Bristol Hospital, Ophthalmic Surgeon and Assistant Physician Accoucher.

Central London Throat Hospital, Three Clinical Assistants.

---

The Duke of Cambridge laid the foundation stone of the new buildings of the London Hospital on Tuesday, when quite a fashionable audience found their way to the East-end. The necessity for the buildings now in course of erection has long been felt. While the hospital (so far as its ward-room accommodation is concerned) has gradually increased since it was first established to hold 130 patients until now, when it holds nearly 800 patients, the department immediately concerned with the first reception of accident and indoor cases has remained much as it was originally.

---

After the ceremony of laying the stone, which was brief, the company went round the wards. A pretty sight occurred in the dining hall, but unfortunately the artist of the *Daily Graphic* was not there. The Hospital Ambulance Corps turned in for tea, which was supplied them by the nurses, and the mingling of the uniforms was very picturesque. The bed dedicated in the children's ward to the memory of Mr. Gerard Wilde attracted much attention; a lovely pot of lilies stood at the foot, and the inmate was a tiny child, such as would have called forth all the tender qualities for which Mr. Wilde's memory is famed.

---

The numerous suicides which have taken place in the medical profession this year point to some great fault surely in the teaching of the schools. Is that a proper education for a young man which sends him forth skilled in surgery and learned in pathology and therapeutics, but without the moral qualities to sustain his mental gifts? Mr. Walter Pearce, of St. Mary's Hospital, who shot himself last week, was a hard worker, aged 36; there was no absolute reason given for his suicide. Dr. Otto Keller, aged 30, took an overdose of morphia on April 9th; he was just making a large and profitable connexion amongst German residents in London. Death claims our medical men so rapidly, we cannot afford that they should themselves seek the end. This week comes the announcement of the death of Mr. Rochfort, at Sydenham, of blood poisoning, contracted in the course of his duties, and we also hear with regret of the death of Dr. Cyril W. Jecks, of University College Hospital, aged only 27, from diphtheria. The last two died at their posts, but those who went before their work was done—who slipped through the open door before the call was given them, must have been wanting in some firmness of will which surely the discipline of a medical school should try to teach. It has too often been urged that the study of medicine has a bad effect on the morals, and some ground is given for this old belief when

"The young and strong who cherish
Noble longings for the strife,
By the roadside fall and perish,
Weary with the march of life."

St. Raphael's Hospital for Consumption issues an annual report and a balance-sheet, for the first time, we believe, and we are glad to welcome this concession by Mrs. Horton. During the year 162 patients have been received, and one has died. Dr. Ayton Gostling has undertaken the duties of hon. medical officer. The income last year was £819, and out of this £214 went to pay off an old debt.

---

The twenty-first Poor Law Conference for the counties of Gloucester, Hereford, Salop, Stafford, Warwick, and Worcester, was opened at the Imperial Hotel, Great Malvern, on May 7th. Mr. F. A. Hyett (vice-chairman of the Gloucestershire Quarter Sessions) presided. Amongst the most interesting papers read were "Classification of Workhouses," by Mr. Murray Browne, "The Poor Law Act, 1889—Deserted Children," by Miss Joanna Hill, and "Boards of Guardians; should they be merged in District Councils?" by Mr. Lloyd Baker.

---

The annual report of Wigan Infirmary says that the amount received during the year has been £10,792. Of this £5,000 was for endowment purposes; and £500 has been transferred to the maintenance reserve account, leaving applicable as ordinary revenue the sum of £5,292. The expenditure for purposes of maintenance has been £6,004, so that the outlay has been £712 more than the amount available for the purpose. The total number of cases dealt with during the year has been 7,014. Of these 1,122 were in-patients, an increase of 31 as compared with 1888-89. Nine hundred and thirty-two were relieved or cured, 30 were discharged unrelieved, 82 died, and 78 remain under treatment at the close of the year. The children's wing is growing rapidly under the builder's hands. The private nursing department has done well, and the income derived from it has been £210.

---

The 23rd annual meeting of the Deaconesses' Institution and Hospital at Tottenham took place on Saturday last. Dr. Laseron (the director) read the annual report of the Council. The average number of patients had been 56, and the average length of their stay in the building, 27 1·3 days. The cost per head was 3s. 0¼d. a-day. In the out-stations at Cork, Dublin, Sunderland, Scarborough, and Jerusalem, the work continued to prosper. The total number of sisters was at present 66, 36 being deaconesses and 30 probationers. During the year 17 probationers had been admitted, but six of them had retired from the work, or been dismissed for ill-health or inefficiency. One sister had died, and six had quitted the institution to enter other spheres of work. The number of patients benefited by the ministrations of the nursing sisters during the year was 12,916.

---

The Derby Corporation have built a new Infectious Diseases Hospital, which was opened on May 1st in the presence of the members of the sanitary committee. At the entrance-gate, Alderman Bemrose presented Mr. Harrison with a golden key, speaking of the time, labour, and attention which the chairman of the committee had bestowed upon the development of the undertaking. Mr. Harrison acknowledged the favour, and the party were admitted, and made a minute inspection of the premises. The greatest admiration was expressed of the whole of the arrangements, and particularly those for the isolation of patients, and the disinfection and purifying of clothing. Mr. Harrison gave the company a history of the ten years' struggle the committee had undertaken in providing the buildings now opened, and congratulated the town on possessing so noble and complete a structure. In the evening the company dined together to celebrate the occasion.

